# Extracts with Nutritional Potential and Their Influence on the Rheological Properties of Dough and Quality Parameters of Bread

**DOI:** 10.3390/foods13030382

**Published:** 2024-01-24

**Authors:** Tatiana Bojňanská, Anna Kolesárová, Matej Čech, Dana Tančinová, Dana Urminská

**Affiliations:** 1 Institute of Food Sciences, Faculty of Biotechnology and Food Sciences, Slovak University of Agriculture in Nitra, Trieda A. Hlinku 2, 949 76 Nitra, Slovakia; anna.kolesarova@uniag.sk (A.K.); matej.cech@uniag.sk (M.Č.); 2 Institute of Biotechnology, Faculty of Biotechnology and Food Sciences, Slovak University of Agriculture in Nitra, Trieda A. Hlinku 2, 949 76 Nitra, Slovakia; dana.tancinova@uniag.sk (D.T.); dana.urminska@uniag.sk (D.U.)

**Keywords:** curcumin, quercetin, Mixolab, dough rheology, Rheofermentometer, baking test, sensory evaluation

## Abstract

Formulating basic food to improve its nutritional profile is one potential method for food innovation. One option in formulating basic food such as bread is to supplement flours with specified amounts of non-bakery raw materials with high nutritional benefits. In the research presented here, we studied the influence of the addition of curcumin and quercetin extracts in amounts of 2.5% and 5% to wheat flour (2.5:97.5; 5:95). The analysis of the rheological properties of dough was carried out using a Mixolab 2. A Rheofermentometer F4 was used to assess the dough’s fermentation, and a Volscan was used to evaluate the baking trials. The effect of the extracts on the rheological properties of dough was measured and found to be statistically significant, with curcumin shortening both dough development time and dough stability. Doughs made with greater quantities of extract had a greater tendency to early starch retrogradation, which negatively affects the shelf life of the end products. The addition of extracts did not significantly affect either the ability to form gas during fermentation or its retention, which is important because this gas is prerequisite to forming a final product with the required volume and porosity of crumb. Less favourable results were found on sensory evaluation, wherein the trial bread was significantly worse than the control wheat bread. The panel’s decision-making might have been influenced by the atypical colour of the bread made with additives, and in case of a trial bread made with quercetin, by a bitter taste. From the technological point of view, the results confirmed that the composite flours prepared with the addition of extracts of curcumin and quercetin in amounts of 2.5% and 5% can be processed according to standard procedures. The final product will be bread with improved nutritional profile and specific sensory properties, specifically an unconventional and attractive colour.

## 1. Introduction

Bread is a staple food, and its consumption, together with that of small wheat pastries, amounts to around 62 kg per year per person in Slovakia [1]. Considering that it is consumed daily and in high amounts, it is essential to pay attention to innovative trends and efforts to formulate recipes that aim to enhance this basic food by improving its nutritional value, possibly using recipes adapted to certain consumers with special needs [2,3,4].

One possible way to make bread and pastries more attractive is the application of non-bakery ingredients to classic bakery flours, the basis of which is wheat. These raw materials bring to final products defined or expected nutritional advantages, and their addition is undoubtedly a promising method for enhancing foods [5,6]. However, the feasibility of using each additive is determined by the amount that must be added, which must be adapted to technological requirements and consumers’ expectations with respect to the innovative additive. The most common benefits include an increase in fibre content and the addition of minerals, substances with antioxidant potential, polyphenolic substances, vitamins, and extracts with specific effects on the human body [7,8,9,10].

Nevertheless, the addition of any non-bakery raw material changes the mode of processing the intermediate products into final products because, depending on the amount of the additive used, systems are created that behave differently from those created by classic bakery ingredients. First, changes in the raw base material manifest in changes in the rheological properties of doughs, water absorption, dough development time, and dough stability [11,12]. For bakeries, the sugar-forming and gas-forming abilities of specific composite flours are important, as is the ability to preserve the dough’s capacity to retain fermentation gases despite gluten dilution by adding gluten-forming, protein-free raw materials [13,14,15,16].

These changes to components of the recipe ultimately cause changes in the final product. By default, the quality of bread is perceived primarily through its volume and sensory properties, and such modified breads are characterized by differences, especially in colour and taste [17,18]. The customer has to be made aware of the change and should perceive it as a benefit and an innovative element.

Flavonoids are plant secondary metabolites that act as natural antioxidants [19] and scavengers of free reactive particles, especially reactive oxygen species. They are inhibitors of lipoxygenases and, by chelating metal ions, prevent their prooxidative action [20]. A positive biological effect of flavonoids on human health has been demonstrated, both *in vitro* and *in vivo*. Antibiotic, anticarcinogenic (chemoprotective), anti-inflammatory, and estrogenic effects have been confirmed. These molecules can act as modulators of enzymatic activity and inhibitors of cell proliferation [21]. In addition, several have a distinctive coloration, which is why they are also used as natural dyes [22].

Quercetin and curcumin are bioactive polyphenolic substances of natural origin that may have positive effects on consumer health when they are consumed regularly [23,24]. Quercetin is a flavonoid found naturally in plant raw materials. It is a yellow-to-green crystalline substance with a bitter taste that is insoluble in cold water, only slightly soluble in hot water, and soluble in fats and alcohols [25]. Quercetin shows a wide range of biological activities. Antioxidant activity has been described as its mode of action in preventing and protecting the liver from non-alcoholic steatohepatitis [26,27], maintaining balance by scavenging free radicals in the human body, protecting DNA from free radicals [28,29], protecting against aging, and maintaining healthy eyesight [30]. Various anticancer effects have also been studied in association with quercetin consumption, especially in cancer of the digestive organs [31,32,33,34,35], but also in cancers of the lungs [36,37,38], breast [39], and colon [40]. The anti-inflammatory effects of quercetin and beneficial effects in diabetes-related complications are also not negligible [41,42,43]. The positive effects of quercetin may also be associated with possible antimicrobial activity [44,45,46,47].

Curcumin is a yellow polyphenol pigment derived from the rhizome of the plant *Curcuma longa* L. that has long been used in cooking and food colouring, but also in medicine [24]. The content of *Curcuma* root may vary, but among the minor components, the most important antioxidants are curcuminoids. More than 50 curcuminoids have been identified, with curcumin, dimethoxy curcumin and bisdemethoxycurcumin being the most abundant [48,49]. The positive effect of curcumin has been studied in the context of various types of cancer [50,51,52,53,54,55], skin problems [56], Alzheimer’s disease [57], cardiovascular diseases (in which it acts by modifying the ratio of HDL to LDL cholesterol) [58], various inflammatory diseases [59,60,61,62], and diabetes-related diseases [63,64]. Curcumin also has antimicrobial properties, which mainly involve penetration of the cell wall, inhibition of cell division and inhibition of the functioning of the cytoplasmic membrane. It also has fungicidal properties and is effective against biofilms [24,65]. These points support its addition to food.

The aim of the presented research was to test the feasibility of applying extracts with nutritional potential, specifically quercetin and curcumin in amounts of 2.5% and 5%, to composite flours and to measure changes in the rheological properties of such doughs and basic quality parameters of the final products.

## 2. Materials and Methods

### 2.1. Material Used

As part of the presented research, two types of non-bakery additives were added to wheat flour: curcumin extract (turmeric extract) from the plant *Curcuma longa*, L. (root) and quercetin dihydrate extract from the plant *Sophora japonica* L. (buds) in amounts of 2.5% and 5%. Particle sizes were characterized by a pass rate through a 100-mesh sieve of 95% for curcumin and a pass rate through an 80-mesh sieve of 95% for quercetin. Losses on drying the extracts were 1.56% (curcumin) and 9.9% (quercetin). The ash content was 0.27% (curcumin) and 0.06% (quercetin). The extracts yielded satisfactory results concerning heavy metal content and microbiological contamination and they are also being used in the pharmacological industry. The extracts were obtained from Natural Field (Xi’an Natural Field Bio-Technique Co., Ltd., Xi’an, China).

Four variants of composite flours and a control flour (wheat flour) were prepared (Table 1). The basic flour used was commercial wheat flour (Mlyn Grznár, Veľké Hoste, Slovakia), the quality of which is defined by the following parameters: energy value (1464 kJ), carbohydrate content (71 g/100 g) including sugars (1.59 g/100 g), fibre content (3.3 g/100 g), protein content (11 g/100 g), fat content (1.3 g/100 g) including saturated fat (0.3 g/100 g), salt (0.01 g/100 g), and ash content (0.65%).

### 2.2. Characterisation of the Antioxidant Ability of Extracts

The solubility of the extracts (0.1–1.0%) was tested in three solvents: demineralized water, 99.8% methanol, and 96% ethanol. Total polyphenol content in the extracts was determined according to a modified version of a method developed by Lachman [66], with Folin-Ciocalteu’s reagent used according to the procedure developed by Komes [67]. The results were expressed as mg/mL of gallic acid equivalents (GAE).

For determination of antioxidant capacity, a method based on free radical scavenging ability by the ABTS (2,2′-azino-bis (3-ethylbenzothiazoline-6-sulfonic acid) radical cation was used [68]. Antioxidant capacity was expressed as mmol/L Trolox equivalents (6-hydroxy-2,5,7,8-tetramethylchroman-2-carboxylic acid).

The ability to reduce or inhibit free radical reactivity was tested by nitrogen radical DPPH (2,2-diphenyl-1-picrylhydrazyl) [68]. The results were expressed as % of scavenging of DPPH radicals.

### 2.3. Mixolab Measurements

Composite flours were analysed for rheological properties using a Mixolab 2 (Chopin Technologies, Villeneuve-la-Garenne, France) according to ICC Standard Method 173 [69]. The Mixolab is used to characterise the rheological behaviour of dough subjected to a dual mixing and temperature constraint. It measures in real time the torque (expressed in Nm) produced by the dough between the two mixing blades, thus allowing the study of rheological and enzymatic parameters.

A Mixolab simulator (S protocol, version 4.05) was specifically developed to make it possible to obtain results comparable (in values and units) to Farinograph data. The conditions for analysis were as follows: constant test temperature, 30 °C; test time, 30 min; running speed, 80 rpm; target value, 1.1 Nm. The parameters evaluated from the Mixolab curves were water absorption (WA), dough development time (DDT), and stability (mixing resistance of the dough).

Mixolab Standard (CH+ protocol, version 4.05) is a tool that allows the complete characterization of a flour (protein, starch, enzyme, etc.) in a single test. It measures the rheological properties of a dough subjected to double stress, namely, kneading and temperature changes. For analysis, a prepared dough of a constant weight of 75 g is hydrated to the desired consistency (1.1 Nm ± 0.05 Nm) during the first phase of testing. The following parameters are evaluated on the recorded curves:

C1 (used to determine water absorption);
C2 (measures protein weakening as a function of mechanical work and temperature);
C3 (measures starch gelatinisation);
C4 (measures high-temperature gel stability);
C5 (measures starch retrogradation in the cooling phase);
slope α (slope of curve between end of period at 30 °C and C2);
slope β (slope of curve between C2 and C3);
slope λ (slope of curve between C3 and C4).

The temperature regime was as follows: temperature 1st level, 30 °C; duration 1st level, 8 min; heating temperature gradient, +4 °C; temperature 2nd level, 90 °C, duration 2nd level, 7 min; cooling temperature gradient, −4 °C; temperature 3rd level, 50 °C; duration 3rd level, 5 min. The total analysis time was 45 min.

Mixolab Profiler is a program that comprehensively characterizes flour (protein network, starch, and enzyme activity), uses a standardized protocol (ICC N°173), and provides a simplified graphical interpretation of the results. It describes flour based on six parameters: absorption potential/Water Absorption Index (which affects dough yield); behaviour in mixing/Mixing Index (which represents the behaviour of the dough during mixing at 30 °C in terms of stability, development time and weakening); gluten strength/Gluten+ Index (which represents the behaviour of the gluten when the dough is heated); maximum viscosity/Viscosity Index (which represents the increase in viscosity during heating and depends on both amylase activity and starch quality); amylase activity/Amylolysis Index (a function of the starch’s ability to withstand amylolysis); retrogradation/Retrogradation Index (a function of the characteristics of the starch and its hydrolysis during the test).

### 2.4. Rheofermentometer Analysis

A Rheofermentometer F4 (Chopin Technologies, France) was used to detect the gas-release kinetics, which differed depending on the composite flours. The following dough parameters were measured: H’m (maximum height of the gas release curve, mm); T’1 (time required to obtain H’m, minutes), Tx (time of porosity, minutes); total volume (total volume of gas produced in mL CO_2_); volume of CO_2_ lost (carbon dioxide volume in mL lost by the dough during proofing), and retention volume (carbon dioxide volume in mL retained in the dough at the end of the test). The method conforms to the AACC 89-01 [70] standard for the measurement of yeast activity and gas production. The proofing chamber was closed hermetically before the series of measurements was begun and was held at 28.5 °C for 180 min.

### 2.5. Breadmaking Procedure

**Figure 1:**
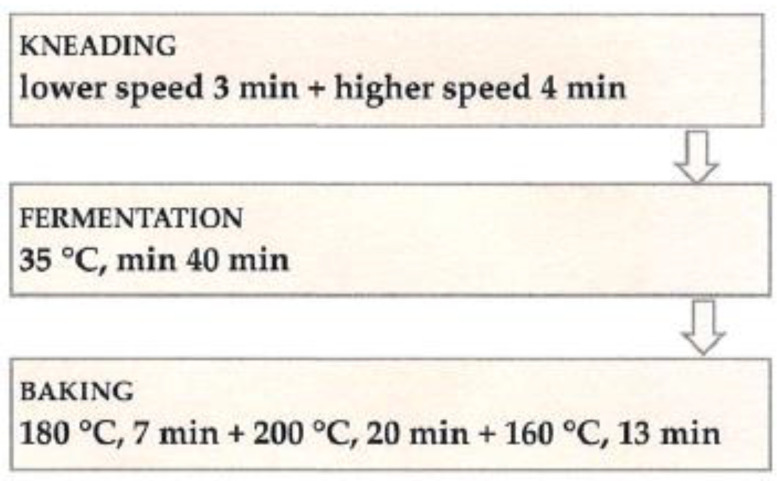
Flow diagram for bread processing.

Wheat flour and composite flour mixed with water were used for baking tests; the hydration of individual dough mixes ranged from 60.7% to 63.2% (determined by the Mixolab, version 4.05). Doughs also included 2.0% NaCl and 1.4% dry yeast. The percentages are based on 100% of the flour mixture. All components were kneaded in a spiral kneader, type SP 12 D (Diosna Dierks & Söhne GmbH, Osnabrück, Germany). After fermentation, the samples were baked with steam (250 mL) (MIWE Condo) (Figure 1). All bread loaves were prepared using the same processing procedure.

### 2.6. Physical Characteristics of Bread

The bread loaves were cooled at room temperature and analysed using a Volscan Profiler volume analyser (Stable Mycrosystems, Surrey, UK) two hours after baking. The following parameters were evaluated: weight of the bread (g), bread volume (mL), specific volume (mL/g), volume yield (mL/100 g flour), and aspect ratio of a middle slice. The laser step was set to 1 mm, and the rotation speed was set to 1 rps. The specific volume was calculated based on loaf volume and mass.

### 2.7. Sensory Evaluation

The experimental loaves were subjected to sensory evaluation by a panel of evaluators (n = 19) using 100-point system in which the following properties were evaluated: overall appearance and shape (1×); surface and properties of the crust (2×); sourness and appearance of the crumb (4×); structure and elasticity of the crumb (4×); smell and taste (9×). The given points of individual properties were recalculated according to their relative importance (indicated in parentheses).

All procedures performed in this study involving human participants were in accordance with the ethical standards of the institutional and/or national research committee and with the 1964 Helsinki declaration and its later amendments or comparable ethical standards.

Bread products were analysed by E-eye analysis using an IRIS VA400 visual analyser (Alpha M.O.S.) with a charge-coupled device camera. The bread samples were placed in the measurement chamber. The collected colour data were represented using IRIS colour codes, which encompass 4096 colours. The analysed data (colour spectrum) were obtained as numerical values of signal intensities.

### 2.8. Presence of Microscopic Fungi

Wheat flour and extracts were analysed for the presence of microscopic fungi. The plate method was used for this analysis. Growth of microscopic fungi was evaluated on Dichloran rose bengal chloramphenicol agar (DRBC; HIMEDIA, Mumbai, India) and Dichloran 18% glycerol agar (DG18; BioLife, Milan, Italy). Plates were incubated at 25 ± 1 °C in the dark for five days. Detected spore-producing filamentous fungi were identified to the genus level based on morphological characteristics according to Samson’s manual [71].

Moulding is the most common cause of spoilage of bakery products. Mould was detected on experimental loaves in two ways: (i) under the same conditions, the experimental breads were cut, and the slices were wrapped in an airtight bag; (ii) the experimental slices of bread were broken under sterile conditions and placed on DRBC culture medium in Petri dishes with a diameter of 150 mm. The samples treated in this way were cultured at room temperature (21 ± 1 °C) for 5 days. Growing colonies were identified to the genus level based on morphological traits according to Samson’s manual [71].

### 2.9. Statistical Analysis

An analysis of variance (ANOVA) and a Duncan’s multiple range test were applied at a significance level of 5% to analyse the significance of the differences between the reference and the samples with different levels of extract incorporated. All analyses were performed in triplicate, and average values were calculated. XLSTAT 2020.5.1 (Lumivero, New York, NY, USA, 2023) [72], together with Microsoft Excel 365 (Microsoft Corporation. Microsoft Excel 365, Redmond, Washington, DC, USA, 2018) [73], were used as the statistical and data analysis software.

## 3. Results and Discussion

### 3.1. Characteristics of Applied Extracts

For our experiment, we added extracts from quercetin and curcumin to composite flours. The curcuminoids curcumin, demethoxycurcumin, and bis-demethoxycurcumin, which are derivatives of 1,7-bis(4-hydroxy-3-methoxyphenyl) hepta-1,6-diene-3,5-dione, are the major secondary metabolites of plants of the genus *Curcuma*. In the food industry, they are mainly used as an intense yellow dye, but recently, significant health benefits of curcuminoids have been demonstrated. These effects are mainly due to the strong antioxidant potential of these substances [58,74]. Quercetin, 2-(3,4-dihydroxyphenyl)-3,5,7-trihydroxy-4H-1-benzopyran-4-one, is a relatively common flavonol in plants that is named after an oak tree of the genus *Quercus*. It has a characteristic bitter taste and is used in the food industry in beverages and as an ingredient in food supplements due to its potential beneficial effects on human health [27,29,35]. Quercetin is an aglycone form of several flavonoid glycosides, e.g., rutin (quercetin-3-O-rutinoside). Curcumin and quercetin are both natural substances with various health effects, as discussed in the introduction.

From the point of view of the bioavailability of biologically valuable substances, the solubility of these substances is very important. As changes in the chemical environment can occur during food preparation (e.g., during dough rising), demineralized water, ethanol (96 wt%), and methanol (99.8 wt%) were used as solvents of the curcumin and quercetin extracts. The results obtained indicate that curcumin extract was completely soluble in methanol up to a concentration of 0.5 wt% and completely soluble in ethanol up to 0.25 wt%. In water, only a colloidal solution formed, and the solution sedimented relatively quickly. Quercetin extract dissolved best in ethanol, with complete solubility up to 1.0 wt%. In methanol, quercetin had complete solubility up to 0.50 wt%, and in water, like curcumin extract, it formed only a colloidal solution.

These findings provide important information to support the application of polyphenol-rich extracts to foods whose preparation involves alcoholic fermentation caused by baker’s yeast. Such processes increase the solubility of curcumin and quercetin extracts. These findings are supported by previous findings [75,76], which confirm that in an aqueous environment, the studied substances dissolve poorly, while alcohol extraction is much more effective for releasing biologically active substances from curcumin and quercetin. This difference is mainly related to the polar nature of most polyphenol compounds [77].

The polyphenol content was determined using the Folin–Ciocaleau reagent, and to ensure fair comparison, the same concentrations of solutions of curcumin extract and quercetin extract were used, namely 0.25 wt%. The total polyphenol content values, presented as gallic acid equivalents, are given in Table 2a. Quercetin extract contained significantly (*p* < 0.05) higher concentrations of total polyphenols than did curcumin with both solvents tested. Higher total antioxidant capacity values, as determined by cationic radical ABTS and expressed in Trolox concentration equivalents, were found for both curcumin and quercetin in ethanol solutions (Table 2b), but the difference was not significant. In methanol solutions, the total antioxidant capacity of quercetin was significantly higher than that of curcumin (*p* < 0.05).

In both solvents, quercetin showed a significantly higher ability to inhibit the reactivity of free radicals, specifically reactive nitrogen species in DPPH, (*p* < 0.05) than did curcumin (Table 2c). The ethanol solution of quercetin extract showed absolute, 100% inhibition of RNS.

### 3.2. Rheological Properties of Composite Flours

**Figure 2:**
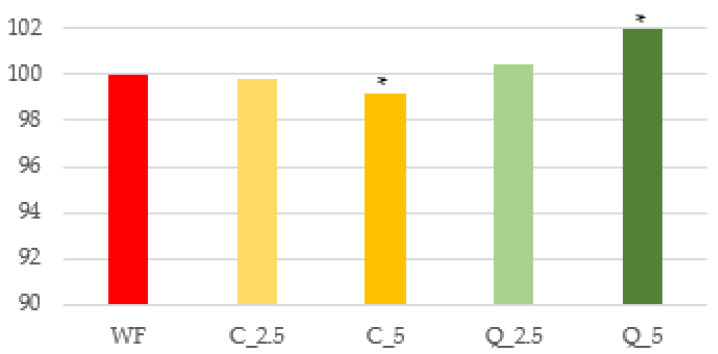
Water absorption of the control flour (100%) and composite flours with extracts. * values are significantly different (*p* < 0.05) compared to the control. WF—wheat flour/control flour; C_2.5—composite flour with 2.5% curcumin extract; C_5—composite flour with 5% curcumin extract; Q_2.5—composite flour with 2.5% quercetin extract; Q_5—composite flour with 5% quercetin extract.

Mixolab was used to determine rheological properties, with tests carried out according to two protocols. The “S” protocol describes the behaviours of the dough during mechanical processing for up to 30 min at room temperature. The first and most fundamental parameter evaluated is water absorption, i.e., the ability of a particular flour to absorb a certain amount of water and obtain an optimal consistency, specifically, 1.1 Nm [78]. The effect of the addition of curcumin and quercetin in amounts of 2.5% and 5% had no significant effect on this parameter, and there is no need to change the mode of preparation and processing of dough to preserve this property. The addition of quercetin tended to increase water absorption, though the difference was significant only at 5% concentration (*p* < 0.05); curcumin, on the contrary, tended to decrease water absorption (Figure 2). This inconclusive effect of quercetin supplementation at 0.05%, 0.1% and 0.5% on water absorption was also found by Lin and Zhou [79]. The low solubility of quercetin in water probably means that it does not compete with other dough components for water during the mixing process.

**Figure 3:**
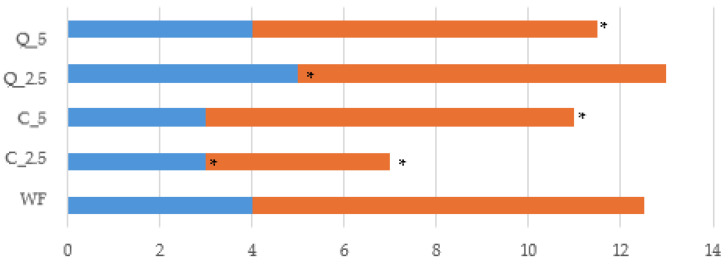
Dough development time and dough stability of the control flour and composite flours with extracts. * values are significantly different (*p* < 0.05) compared to the control. WF—wheat flour/control flour; C_2.5—composite flour with 2.5% curcumin extract; C_5—composite flour with 5% curcumin extract; Q_2.5—composite flour with 2.5% quercetin extract; Q_5—composite flour with 5% quercetin extract.

In terms of dough development time (DDT) and dough stability (DS), the composite flour doughs most similar to the control were those with quercetin was added in amounts of 2.5% and 5%. Other authors have even found a prolonged DDT with the addition of quercetin to almost 20% [79]. Quercetin apparently reacts with gluten, which negatively affects protein hydration and causes a longer dough development time, a change that may be associated with a loss of its elasticity due to alterations in the dough structure [80]. In our samples, the addition of curcumin significantly (*p* < 0.05) shortened DDT compared to that of the control, with differences up to 25% (one minute), but it also affected stability. The stability of the dough with the addition of 2.5% curcumin was significantly (*p* < 0.05) lower than that of the control dough and of doughs with the addition of quercetin (Figure 3).

The changes in the properties of composite flour also depend on the form of additive used. The addition of curcumin extract (more than 95% curcuminoids) in our research caused a slight decrease in water absorption; however, Park, Lin, and Hwang [81] found an increase in water absorption after adding turmeric powder in amounts of 6% and 8%, a change that was also associated with DDT prolongation and DS shortening.

**Figure 4:**
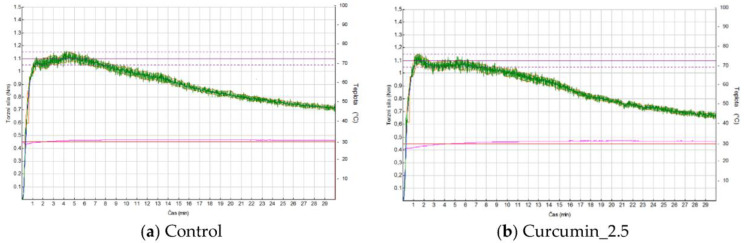
(**a**,**b**) Mixolab S protocol/Farinographic curves of the control flour and composite flour with curcumin extracts. (**a**) Control—wheat flour, (**b**) Curcumin_2.5—composite flour with 2.5% curcumin extract.

The curve showing the tension of the kneading dough decreased significantly less (*p* < 0.05) for the doughs with the addition of 5% curcumin and 5% quercetin than for the control, and these doughs can therefore be considered weaker. Only minimal differences were measured between the curves for the 2.5% extracts and the control. These results are comparable to those obtained using a Farinograph in study of Hadnađev et al. [82] with the “S” protocol and can be taken to indicate a promising product that does not require additional technological inputs. Between these products, the greater difference in DDT and DS compared to the control was found in the curves for the dough with 2.5% curcumin (Figure 4a,b). However, even from the graphs, it is clear that there was no problematic deterioration of rheological properties. Based on these results, we do not expect the addition of extracts to cause a significant deterioration in the dough’s handling characteristics during commercial baking processes.

The “CH+” protocol includes measures of the characteristics of dough during mixing, heating, and cooling. The output is a curve based on the changes in torque with time and temperature. The shape of this curve predicts the suitability of the dough for a given food use, provides information of the projected baking characteristics of the dough, and yields quality profiles of both proteins and starches [83].

**Figure 5:**
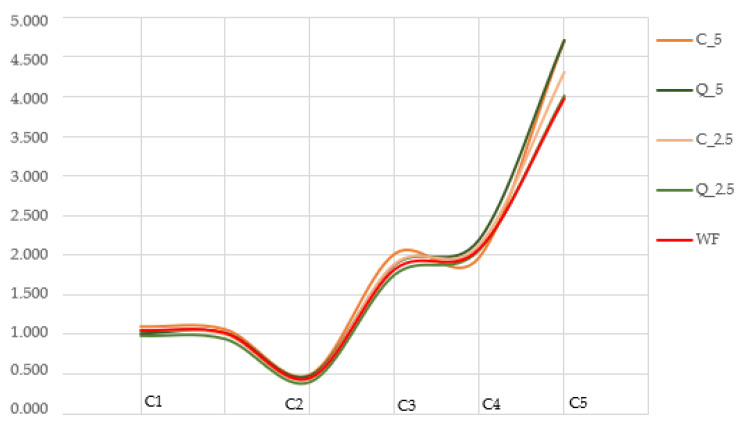
Rheological behaviour curves (CH+ protocol, Mixolab). WF—wheat flour/control flour; C_2.5—composite flour with 2.5% curcumin extract; C_5—composite flour with 5% curcumin extract; Q_2.5—composite flour with 2.5% quercetin extract; Q_5—composite flour with 5% quercetin extract. C1—maximum torque during mixing; C2—weakening of the protein; C3—rate of starch gelatinization; C4—minimum torque during heating; C5—torque after cooling at 50 °C.

**Figure 6:**
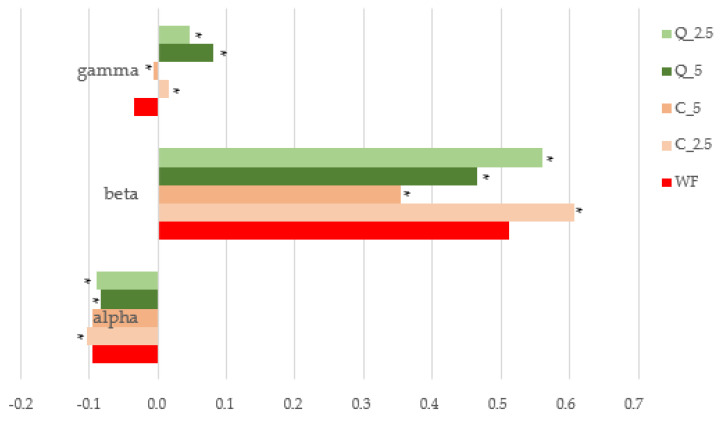
Mixolab—calculated parameters: alpha, beta, and gamma slopes. * values are significantly different (*p* < 0.05) compared to the control. WF—wheat flour/control flour; C_2.5—composite flour with 2.5% curcumin extract; C_5—composite flour with 5% curcumin extract; Q_2.5—composite flour with 2.5% quercetin extract; Q_5—composite flour with 5% quercetin extract.

No differences were observed between the evaluated samples at the beginning of the analysis (1st level) in the C1 and C2 point ranges. However, with increasing dough temperature (2nd level, points C3, C4), doughs with added curcumin and quercetin began to behave differently from control wheat dough, as in the case of dough cooling (3rd level, point C5) (Figure 5). The behaviour of doughs at increasing temperatures is shown by slope α (Figure 6), which reveals that quercetin-added doughs show faster protein weakening at high temperatures. Furthermore, increasing temperatures up to 90 °C caused differences in starch gelatinization in the doughs, with these differences expressed as slope β. The addition of lower amounts of curcumin and quercetin significantly (*p* < 0.05) increased the value of slope β (Figure 6) compared to that of wheat dough; however, when greater amounts were added (5%), the values were consistently lower than those for the control dough. Starting at a specific temperature, the phenomena linked to starch gelatinization become dominant, resulting thereafter in increased viscosity. The intensity of this increase depends on the quality of the starch and, in some cases, on the additives [84,85]. The highest value of torque in point C3 was observed in a sample of dough with the addition of 5% curcumin.

It has been found that the rheological profile of non-heated and heated starch dispersions is affected by the content of damaged starch and the granular size profile, which could be useful in flow-regime studies, processing of variables, and the evaluation of final product properties [84,86]. Various studies have been carried out on wheat flour to understand how the proteins and starches change when they are subjected to mixing and temperature constraints, but structural changes in the proteins and starches at the moisture levels typical of a dough system are not fully understood [87].

The value of viscosity (torque) at the end of the plateau (at the end of the analysis) depends considerably on the activity of endogenous or added amylases. The greater the decrease in viscosity, the greater the amylase activity. The enzymatic degradation speed, expressed as slope λ (Figure 6), was lowest in the control dough, and the addition of quercetin increased this value significantly (*p* < 0.05) more than the addition of curcumin did. The final torque value at point C5 was higher in composite flour doughs in the following ascending order: control (3.321 Nm) < quercetin_2.5% (4.012 Nm) < curcumin_2.5% (4.309 Nm) < quercetin_5% = curcumin_5% (4.708 Nm). As the doughs cool down, starch retrogrades and product viscosity increases. Some additives influence this phenomenon and limit its importance, thus delaying staling and ensuring that the finished product is softer [88].

In the case of the addition of curcumin and quercetin, the opposite phenomenon has been observed, and the addition of these polyphenol-rich products does not have a potentially positive effect on prolonging the freshness of the bread. A number of studies have aimed at slowing the aging of bread through the addition of various natural ingredients, and among the most studied are various polysaccharides [89,90,91,92]. Another option is the use of leaven. Torrieri et al. [93] observed that the addition to bread dough of 30% fermented leaven with lactobacilli forming exopolysaccharides positively affected the rates of moisture loss and retrogradation of starch.

Cho et al. [94] examined dough behaviours following the addition of rutin, which is chemically related to quercetin. They found that during the heating cycle, torque values changed significantly; at point C2, they were reduced, which is associated with a greater rate of protein attenuation. However, with increasing concentrations of added rutins, torque values at points C3 and C5 decreased, a change associated with a lower degree of starch gelatinization and retrogradation. Other authors [82,95] report that Mixolab parameters C2, C3, C4 and C5 are significantly correlated with bread volume. Significant correlations have also been observed between the value of slope α and Zeleny sedimentation, Alveograph results, Farinograph softening degree, and stability values.

**Figure 7:**
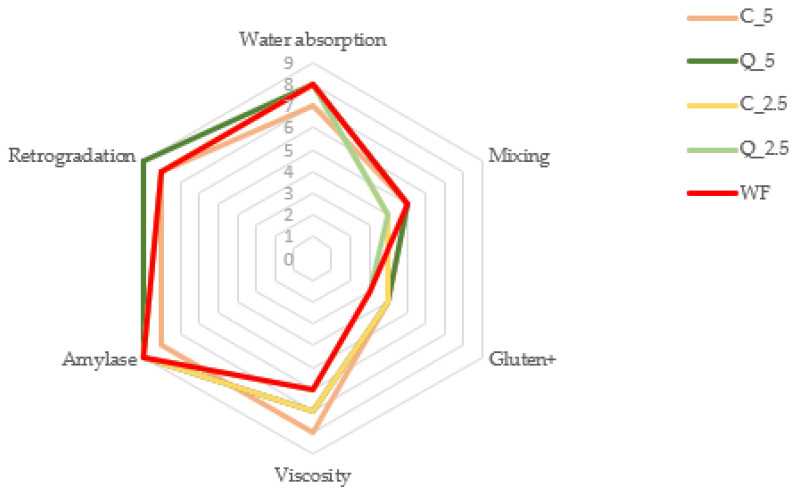
Mixolab Profiler-generated complete characterization (protein network, starch, and enzyme activity) of control flour and flour with the addition of extracts according to fundamental criteria: water absorption index, mixing index, gluten+ index, viscosity index, amylase index and retrogradation index. WF—wheat flour/control flour; C_2.5—composite flour with 2.5% curcumin extract; C_5—composite flour with 5% curcumin extract; Q_2.5—composite flour with 2.5% quercetin extract; Q_5—composite flour with 5% quercetin extract.

Figure 7 shows the Mixolab Profiler results for evaluated doughs, which can serve as an illustrative graph of a comprehensive evaluation, with all observed results expressed in terms of practical uses of the dough. The six criteria/indices calculated by the Profiler program comprehensively describe the quality of the flours [82,83]. If we consider control wheat flour as the quality standard, even small additions of curcumin and quercetin change technologically important properties of the dough, especially the temperature stability of the protein (gluten) and viscosity. The size of the starch grains and the viscosity of the dough increase (between points C2 and C3) as the dough passes from a gluten-based system to a starch-based system. The greatest increase in viscosity was in samples with the addition of curcumin (1.528 Nm and 1.443 Nm), and the sample most similar to the control was the sample with the addition of 2.5% quercetin (1.820 Nm and 1.760 Nm). The last index evaluates the initial stage of amylose retrogradation and is a suitable indicator of total crystallization of starch in flour. During cooling (3rd level) of gelatinized starch, swollen or damaged starch grains tend to restore the crystalline structure through starch retrogradation, which determines the shelf life of the product. This issue has been thoroughly studied, and mathematical models capable of predicting the kinetics of starch retrogradation in bread during storage have been developed by Del Nobile et al. [96]. Possible methods of influencing this process are being studied by Arp et al. and Hayes et al. [97,98]. Our results confirmed that dough with the addition of 5% quercetin exhibited the greatest tendency towards early retrogradation.

### 3.3. Ability of Experimental Doughs to Form and Conserve Fermentation Gases

**Figure 8:**
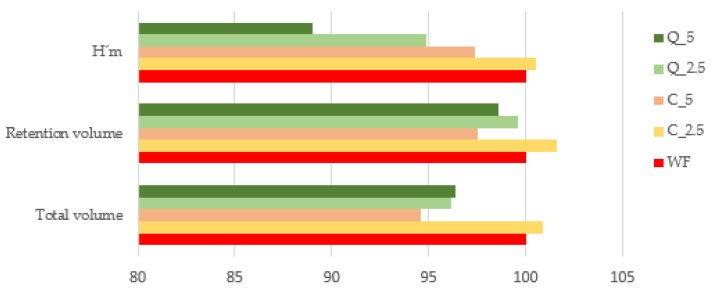
Rheofermentometer—evaluation of dough behaviour during fermentation according to fundamental criteria: H’m—maximum height of the gas-release curve; total volume—total volume of gas produced in mL CO_2_; retention volume—CO_2_ volume in mL retained in the dough at the end of the test. WF—wheat flour/control flour (100%); C_2.5—composite flour with 2.5% curcumin extract; C_5—composite flour with 5% curcumin extract; Q_2.5—composite flour with 2.5% quercetin extract; Q_5—composite flour with 5% quercetin extract.

Rheofermentometer analysis of flour and dough enables accurate simulation of processing conditions during the production of baked goods containing yeast. Figure 8 shows the differences between the evaluated experimental doughs during the fermentation process under laboratory conditions using a Rheofermentometer. The decrease in the fermentation ability of doughs with the addition of extracts of curcumin or quercetin in small amounts (2.5% and 5%) is generally not significant. The addition of 2.5% curcumin even increased the volume of fermented gas produced (result not significant). The addition of more curcumin and the addition of quercetin caused a decrease in the amount of fermentation gas produced, but the maximum decrease compared to the control wheat flour was only 5.8%. This decrease was observed in the sample with the addition of 5% quercetin.

Nevertheless, it is not only the amount of gas produced that is important, but also how much of that gas the dough can capture in its gluten structures under the conditions of analysis in 180 min. This value is positively correlated with the volume of bread after baking [93], so we consider it an important indicator of technological quality. Again, we can conclude that the additions of the evaluated extracts at 2.5% and 5% did not significantly affect the gas retention of the doughs compared to the control dough, although in the case of the addition of 2.5% curcumin, there was an increase in retention volume compared to the control. The retention volume was very slightly decreased by additions of the evaluated extracts at 5%.

H’m is the maximum height of the gas-release curve, and together with T’1 (time required to obtain H’m), this value gives us important information about the rate and intensity of formation of fermenting gases. The addition of 5% quercetin had a negative effect on this parameter, while the addition of curcumin had only a non-significant effect.

**Figure 9:**
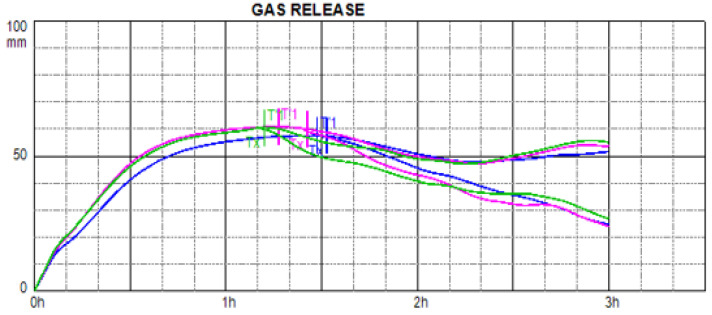
Rheofermentometer curve of control dough and dough with addition of 2.5% extract. Green—control/wheat dough; purple—2.5% curcumin; blue—2.5% quercetin.

From Figure 9, it is clear that the curves showing the rheofermentographic behaviours of doughs with additions of 2.5% extract almost completely follow the control-dough curve (green colour). A slight difference is detectable in the curve for the dough with the addition of 2.5% quercetin (blue colour), which shows slower gas formation. Based on the results obtained, the addition of both extracts in both amounts result in doughs that can be characterized as acceptable, and these extracts do not cause significant changes in the gas-forming ability of flours. The only significant (*p* < 0.05) differences were found in T’1, i.e., time required to obtain the maximum height of the gas release curve H’m. The addition of 2.5% quercetin prolonged this time, while the addition of 5% curcumin shortened it. These results were subsequently seen to be related to the later onset of dough porosity time, which is when the dough starts to lose CO_2_. In the end, however, this difference did not affect the retention volume. Hwang et al. [99] found that with the increasing addition of quercetin, CO_2_ production decreased. They attribute this change to its undesirable effect on yeast activity through antifungal effects and specifically to inhibition of enzymes responsible for cell-wall formation. In addition, both quercetin and curcumin limit yeast growth due to their capacity for iron chelation, resulting in less fermentation gas being produced in the dough [100,101]. Other authors have also confirmed the undesirable effect of quercetin on gas-retention capacity, which leads ultimately to lower bread volume [79,102].

The impact of various gas production rates on the time of dough porosity Tx and the corresponding CO_2_ volume were studied by Verheyen et al. [103], and Codina et al. [104] assessed the correlation between the fermentable sugar content of dough and its behaviour during fermentation. In general, most non-bakery additions reduce the volume by retention, primarily due to gluten dilution, but Babin et al. [105] concluded that gas retention capacity is less affected by dough composition than by current aeration state. However, the addition of legume flour decreased retention volume with higher additions (10% or more) [93], as well as in the case of adding milk thistle flour, spelt wheat flour, amaranth flour, and buckwheat flour [90,106].

### 3.4. Bread Evaluation

Trial breads were evaluated based on basic technological parameters, among which bread volume is undoubtedly of the greatest importance. This is the indicator that is also most crucial from the sensory point of view, and consumers generally expect bread with a large volume and reasonably porous crumb. The volume of bread is the result of the expansion of the trapped fermentation gases formed during both souring and evaporation, when the volume is additionally increased and the dough structure changes irreversibly [107].

Bread weight is also interesting from an economic point of view, but due to the approximately equal water absorption of all composite flours, no differences in the weight of the breads were found.

**Figure 10:**
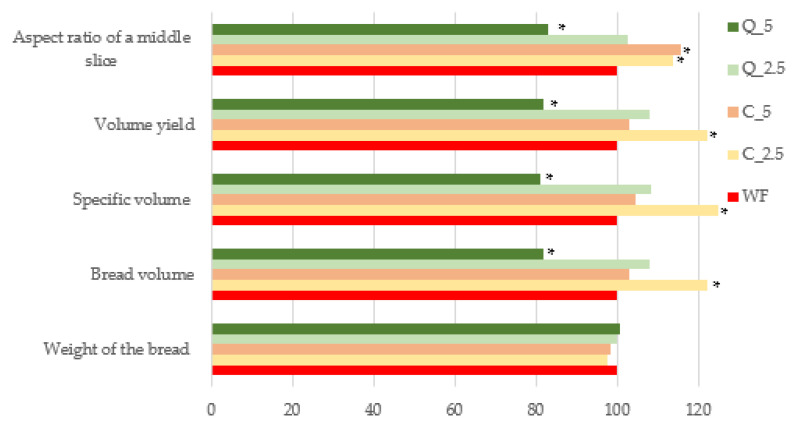
Baking experiment—evaluation of the technological quality of trial breads by Voscan. * values are significantly different (*p* < 0.05) compared to the control. WF—control/wheat flour/control loaf (100%); C_2.5—composite flour with 2.5% curcumin extract; C_5—composite flour with 5% curcumin extract; Q_2.5—composite flour with 2.5% quercetin extract; Q_5—composite flour with 5% quercetin extract.

The test breads differed from the control in three key parameters (bread volume, specific volume, and volume yield) that are related, with the degree of difference depending on the additive and its amount. Both the 2.5% curcumin and 5% curcumin doughs were found to yield better results than control doughs, with the best result seen with the addition of 2.5% curcumin (Figure 10). This was an interesting finding, but given the previous results for the rheological and gas-forming properties of composite flours, it was not surprising; the dough with the addition of 2.5% curcumin had the greatest ability to create and retain fermentation gases. The addition of quercetin to bread (at concentrations of 1.2%, 2.4%, and 3.6%) has been shown to reduce bread volume and lead to deterioration of bread texture in research by other authors [79,102]. Deterioration in the dough’s rheological properties and the smaller volumes of bread are due to plant polyphenols, as described by [108,109,110,111]. Nevertheless, the additives tested in our research can be considered acceptable in the amounts tested, except for the quercetin at 5%.

**Figure 11:**
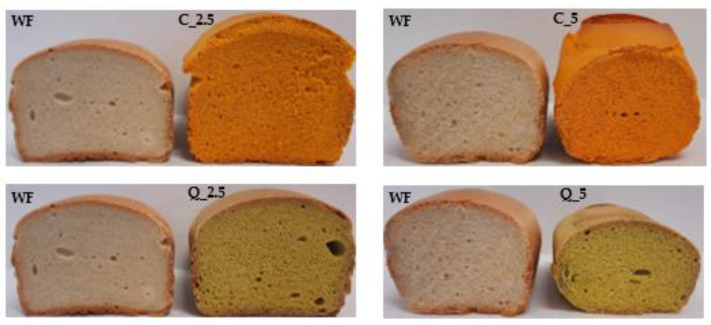
Baking experiment—photographs of trial breads. WF—wheat flour/control flour; C_2.5—composite flour with 2.5% curcumin extract; C_5—composite flour with 5% curcumin extract; Q_2.5—composite flour with 2.5% quercetin extract; Q_5—composite flour with 5% quercetin extract.

**Figure 12:**
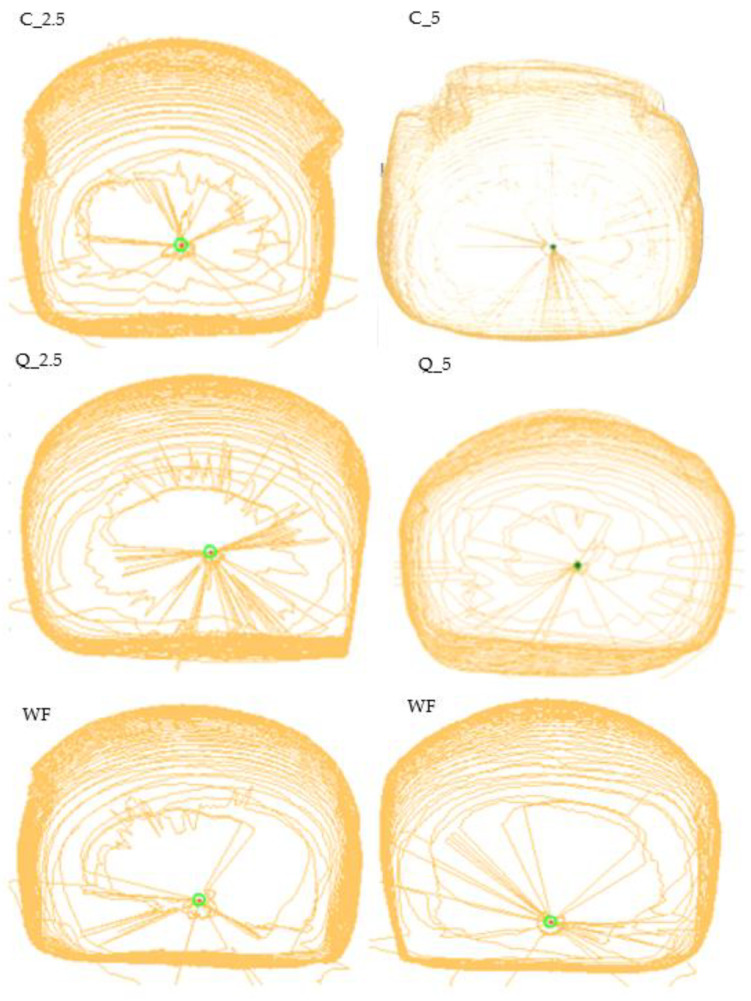
Baking experiment—graphic scans of trial breads by Volscan. WF—wheat flour/control flour; C_2.5—composite flour with 2.5% curcumin extract; C_5—composite flour with 5% curcumin extract; Q_2.5—composite flour with 2.5% quercetin extract; Q_5—composite flour with 5% quercetin extract.

The arching of the loaves (aspect ratio) was also evaluated; as with the previous indicators, except for the loaf with the addition of 5% of quercetin, all experimental loaves were better arched than the control. This result is also documented by photographs of baked loaves compared to control loaves (Figure 11). The objective parameters were measured by Volscan (Figure 12).

**Figure 13:**
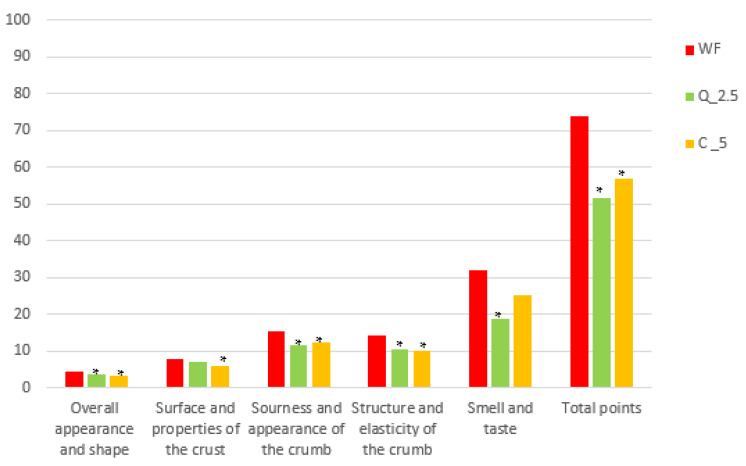
Baking experiment—sensory evaluation of the trial breads by means of a 100-point system. * values are significantly different (*p* < 0.05) compared to the control. WF—wheat flour/control flour; C_5—composite flour with 5% curcumin extract; Q_2.5—composite flour with 2.5% quercetin extract.

Sensory evaluation is very important when assessing food quality in general (and in research and development of enhanced products) because ultimately it is consumers who decide whether an novel product is suitable and consumable. Based on a preliminary (rapid) sensory evaluation, variants (2.5% of quercetin and 5% curcumin) were selected for the baking trial, in which we assumed they could be accepted by consumers. However, the results of the sensory assessment, expressed as points on a 100-point scale, did not meet our expectations. Both bread with added quercetin and bread with added curcumin were overall rated significantly (*p* < 0.05) worse than control wheat bread (Figure 13). Only in terms of bread surface and crust was bread with the addition of 2.5% quercetin rated as well as control bread. More importantly, in terms of the smell and taste of bread, bread with the addition of 5% curcumin was rated as well as the control, which is a promising finding, as similar results were observed in other sensory evaluations [109].

The previously cited authors [79] who added quercetin to bread also found deterioration in the sensory properties of breads with increasing concentrations of quercetin (crust hardness, chewing of the crumb, volume, porosity). It is a fact that non-bakery raw materials in most cases worsen the technological parameters of bread, and it is necessary to establish optimal amounts of additives [111,112,113]. Excessively high concentrations can cause problems; however, breads with lower concentrations of additives may even be rated better than control breads [114].

Although the results of the sensory analysis of our experimental loaves with quercetin and curcumin confirmed the trend toward worse overall sensory properties of experimental breads made with the addition of various bioactive substances, the overall results were relatively satisfactory and offer some hope for future developments. It should also be pointed out that with a higher additive concentration, the content of biologically active substances increases, thus increasing the health benefits discussed above. For this reason, it is essential to strike a balance between the satisfactory sensory and technological properties of new and innovative bakery products and their nutritional benefits. In food production, modern procedures (e.g., co-crystallization and encapsulation) that affect the physicochemical properties of compounds (solubility, bioavailability, and biostability) could also be used to mask the undesirable sensory attributes of quercetin and curcumin [115,116,117]. Bojňanská et al. [118] describe possible impacts on production technology and price increases associated with innovative bakery products, and based on the above findings, it can be assumed that the addition of extracts in low amounts of 2.5% and 5% do not have a limiting effect in terms of cost.

The main differences between the wheat bread and the breads made with the addition of extracts were the colours of the crust and especially of the crumb. As is evident from Figure 11, the colours of both breads were very unconventional, which can pose a problem, especially for conservative consumers.

**Figure 14:**
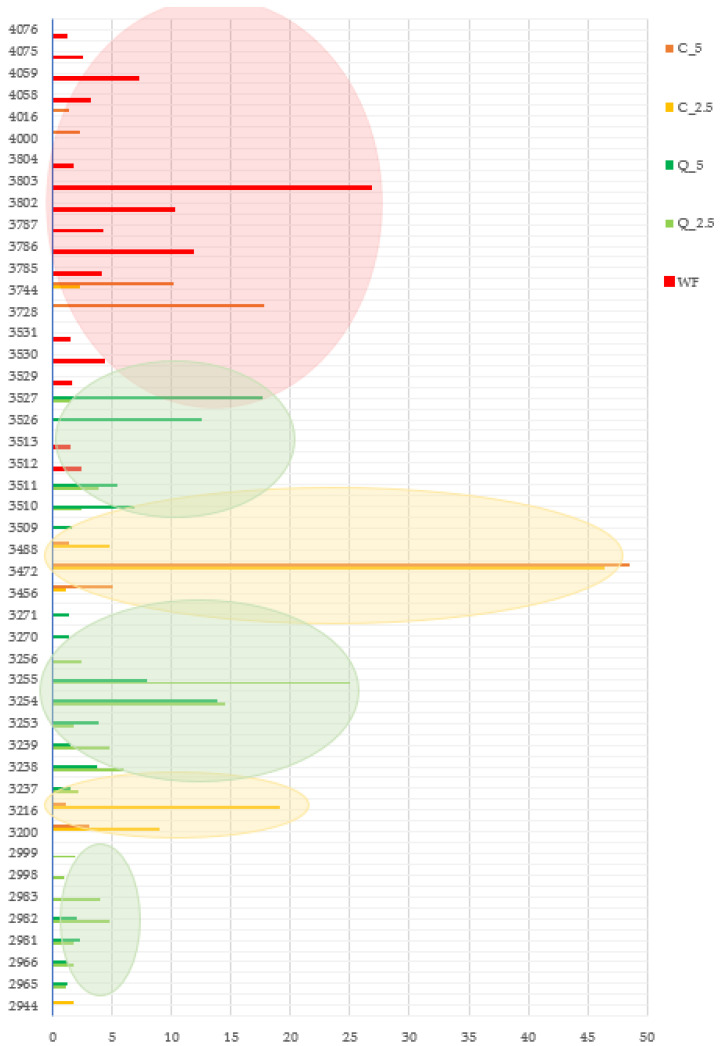
Baking experiment—colour spectra of trial breads evaluated by E-eye. 2944–4076—colour shades. WF—wheat flour/control flour; C_2.5—composite flour with 2.5% curcumin extract; C_5—composite flour with 5% curcumin extract; Q_2.5—composite flour with 2.5% quercetin extract; Q_5—composite flour with 5% quercetin extract.

The complete colour spectrum of the breads was analysed by instrumental sensory analysis using an E-eye, and the results are graphically shown on Figure 14. There were fundamental differences between the samples in terms of colour. In the control bread, 15 colour shades were detected, which together defined the colour of standard bread. The simplest colour was seen in bread made with the addition of curcumin, in which there were two colour shades; between these, one colour was significantly dominant. In total, only seven colour shades were identified. Bread made with the addition of quercetin was more colourful, with 17 colour shades that could be divided into three groups, two of which were more dominant.

The addition of natural dyes to baking products mainly results in a change in the colour of the crumb, while the colour of the crust is more influenced by changes associated with the Maillard reaction [119]. Non-traditional breads may be less attractive to more conservative consumers, but we believe that on the other hand, an atypical colour can make bread more attractive and interesting for more progressive (courageous) consumers. Although the addition of curcumin powder to regular wheat bread worsens its textural properties and decreases its volume, it also increases antioxidant activity and decreases water activity, so the shelf life of the bread can potentially be extended. Lim et al. [109] suggested that this advantage, together with the unconventional colour, make the bread interesting.

#### Presence of Microscopic Fungi

The shelf life of bread is associated with possible microbiological risks. Flour is an essential raw material in breadmaking and can be a source of microscopic fungi that can get into the air during production and contaminate finished products [120]. In mycological analysis of the flour used for the analyses, a very low level of contamination of <4102 KJT/g was detected on both DRBC and DG18. When the applied quercetin and curcumin extracts were analysed, no colonies of mould were grown.

Bread is one of the most important staple foods in the world and can be spoiled by many microscopic filamentous fungi (moulds). The spectrum of moulds that cause spoilage of bread is influenced by the type of bread and the storage temperature [121]. As consumers often store bread in sealed containers, such storage was modelled in our experiments; moulds were grown on trial bread stored in sealed bags. We simultaneously evaluated mould growth on agar (Dichloran rose bengal agar) medium in petri dishes.

Mould contamination of bread and bakery products is a cause for concern and generates economic losses and consumer dissatisfaction [122]. Pateras et al. [123] report that a wide variety of moulds occur in wheat bread, including *Penicillium*, *Aspergillus*, *Cladosporium*, Mucorales, and *Neurospora*. Most often, species of the genus *Penicillium* are involved in mould on bread [121,124], and this finding was also confirmed in our research.

The results of our findings are presented in Table 3 and show that the most frequently occurring genus was *Penicillium* (9/12), followed by *Alternaria* (3/12). Other identified genera of mould were present at lower abundances (1–2/12). Based on these results, it is not yet possible to make an unequivocal statement regarding the impact of the added extract on the possible prolongation of safe storage, although this possibility has been proposed [24,44,46,47,65].

Storing trial bread samples on DRBC medium allowed more mould to grow and be identified than was seen on the same trial breads packed in sealed bags. We explain this difference by the fact that the medium provided the fungi with nutrients and promoted rapid growth; in closed bags on gradually aging and drying breads, the spores did not germinate. Nevertheless, we found that samples with higher levels of extract had a higher incidence of microscopic mould than did controls and breads with lower levels of extract, despite the fact that no growing mould colonies were observed in the analysis of the quercetin and curcumin extracts. It will be essential to further examine this issue.

In the following paragraphs, we consider benefits as well as challenges in the production of innovative food with the addition of non-bakery raw materials. Based on the results of the presented research, there is a legitimate question as to whether such a procedure in the production of bread as an everyday food is feasible, and it is also necessary to evaluate the benefits and disadvantages of using plant extracts in modified foods.

The main benefits can be attributed to certain expected nutritional benefits, but here it is still necessary to consider changes in the content of valuable ingredients caused by production of a particular food. For example, the addition of curcumin to a selected cereal product can improve the antioxidant activity, as expressed by a DPPH scavenging activity [125], but on the other hand, the high temperatures required for bread production cause degradation of curcumin [126], so the expected nutritional effects may not materialize. On the other hand, according to Borghetti et al. [127], the thermal stability of quercetin is higher and depends mainly on the degree of hydration. The authors point out that quercetin dihydrate is the most heat-resistant form of quercetin, and this is also the form that was used in our research.

Quercetin is also usable, even in small amounts, as a functional ingredient in cereal products, to provide consumers with increased antioxidant intake and the associated benefits, as discussed above. At the same time, the addition of quercetin to bakery products is associated with high inhibition of fluorescent advanced glycation end products (AGEs) via several different mechanisms [79,102]. AGEs are ingested primarily in the diet, wherein they arise from the Maillard reaction. These substances have the ability to bind to cell receptors, resulting in aberrant metabolism, the release of inflammatory cytokines, and ultimately to worsening of diabetes [128]. At the same time, the glycation process produces a significant amount of reactive oxygen systems, resulting in significant oxidative stress. Research has shown that quercetin can prevent oxidative stress by inhibiting the protein-glycation process through its antioxidant properties [129,130]. However, its great sensory disadvantage is its bitterness, which is very difficult to mask in cereal products in ordinary ways.

The solvable disadvantages of bread made with the addition of non-bakery raw materials are changes in the technological properties of intermediate products, including in the rheological and rheofermentographic properties of dough, and changes in the final product both in technological (volume of bread) and sensory (colour, taste, etc.) terms. It is necessary to conduct further research and find answers to other important questions.

## 4. Conclusions

Flavonoids are biologically valuable substances, and their intake through foods of daily consumption could potentially have a positive effect on human body. This research aimed at testing the feasibility of adding the plant extracts curcumin and quercetin to composite flours and evaluating their impact on the properties of intermediate products and the final product, a trial bread. Based on the results of our experiments, we can offer several conclusions.

From a food point of view, the solubility of the applied extracts is important. Neither curcumin nor quercetin is soluble in water. They were found to be completely soluble in ethanol and methanol up to specific concentrations; specifically, in methanol, both curcumin and quercetin were completely soluble up to a concentration of 0.5 wt%, while in ethanol, curcumin was completely soluble up to a concentration of 0.25 wt% and quercetin was completely soluble up to a concentration of 1.0 wt%.

Total polyphenol content, total antioxidant capacity and % of scavenging of DPPH of the tested extracts were determined and compared with each other, and in terms of these results, quercetin can be considered more valuable. However, its confirmed disadvantage is its bitter taste even at low concentrations and, for basic cereal foods, an atypical green colour. The second evaluated extract, curcumin, can also be considered a valuable component of enhanced foods.

When extracts are added to flour that will be processed into dough, it is important from a technological point of view to know the rheological properties of the intermediate products. The effect of extracts on dough development and stability was measured and statistically confirmed, with curcumin shortening both dough development time and dough stability and quercetin having less significant effects. The doughs were both workable but were characterized by distinctive colours (yellow and green). By evaluating the characteristics of the doughs during mixing, heating, and cooling, it was possible to predict their suitability for bread production and the shelf life of bread products made with these extracts. Compared to the control wheat dough, differences in protein weakening, starch gelatinization and retrogradation were found in doughs made with the addition of extracts. These differences became apparent when the baked bread reached high temperatures and during subsequent cooling. Doughs with higher extract concentrations also had a greater tendency to early starch retrogradation.

The addition of extracts did not significantly affect the ability of the doughs to form gas during fermentation or their ability to retain the formed volume of gas, which is promising in terms of their ability to yield a final product with the required volume and porosity of crumb. The best results were found for dough made with the addition of 2.5% curcumin, and this result was confirmed by the results of the baking experiment. By contrast, the worst trial loaf was the one made with the addition of 5% quercetin. Nevertheless, all tested doughs were rated acceptable from a technological point of view. The results of the sensory evaluation were less favourable. The trial bread was significantly worse than the control wheat bread. The panel’s decision-making might have been influenced by the atypical colour of the bread and the bitter taste of the trial bread made with quercetin.

When concentrated, valuable ingredients are added to basic foods, the advantages come from the nutritional benefits, and the disadvantages consist of the changes in technological properties, which often require modifications in technological processes. In the case of the addition of extracts of curcumin and quercetin in amounts of 2.5% and 5%, the results confirmed that the composite flours can be processed according to standard procedures, with the final product being bread with specific sensory properties, especially an unconventional and attractive colour.

## Figures and Tables

**Table 1:** Formulations using the evaluated composite flour (experimental design).

Wheat Flour T650	Curcumin	Quercetin	Identification of the Sample	
100%	0%	0%	WF	
97.5%	2.5%	2.5%	C_2.5	Q_2.5
95%	5%	5%	C_5	Q_5

WF (wheat flour); C_2.5 (curcumin 2.5%); C_5 (curcumin 5%); Q_2.5 (2.5% quercetin); Q_5 (quercetin 5%).

**Table 2:** (**a**) Total polyphenol content. (**b**) Total antioxidant capacity. (**c**) Percent scavenging of DPPH.

Extract	Methanol Solvent	Ethanol Solvent
a.		
Curcumin 0.25%	1.389 ± 0.016 a	1.289 ± 0.008 a
Quercetin dihydrate 0.25%	2.656 ± 0.020 b	3.274 ± 0.034 b
b.		
Curcumin 0.25%	2.890 ± 0.042 a	3.081 ± 0.080 a
Quercetin dihydrate 0.25%	3.045 ± 0.039 b	3.118 ± 0.023 a
c.		
Curcumin 0.25%	94.43 ± 0.72 a	87.32 ± 0.46 a
Quercetin dihydrate 0.25%	96.57 ± 0.53 b	100.0 ± 0.01 b

Values followed by the same letter in the column are not significantly different from each other (*p* > 0.05).

**Table 3:** The appearance of microscopic fungi on trial breads.

Identified Mould	DRBC					
	WF1	Q_2.5	C_2.5	WF2	Q_5	C_5
*Penicillium* sp.	+	+		+	+	+
*Alternaria* sp.				+		+
*Nigrospora oryzae*					+	
*Aspergillus* (form *Eurotium*)					+	
*Aspergillus* sp.						
*Aspergillus* Sektion *Circumdati*						
*Aspergillus* Sektion *Nigri*						+
*Epicoccum nigrum*						+
*Chaetomuim* sp.			+			
*Cladosporium* sp.			+			
	**In a Closed Package**					
	**WF1**	**Q_2.5**	**C_2.5**	**WF2**	**Q_5**	**C_5**
*Penicillium* sp.		+	+		+	+
*Alternaria* sp.				+		
*Nigrospora oryzae*						
*Aspergillus* (form *Eurotium*)					+	
*Aspergillus* sp.	+		+			
*Aspergillus* Sektion *Circumdati*					+	
*Aspergillus* Sektion *Nigri*						+
*Epicoccum nigrum*						
*Chaetomuim* sp.						
*Cladosporium* sp.						

DRBC—Dichloran rose bengal agar, +—The genus has been qualitatively identified, WF1, WF2—control bread; C_2.5—bread with curcumin 2.5% extract; C_5—bread with curcumin 5% extract; Q_2.5—bread with quercetin 2.5% extract; Q_5—bread with quercetin 5% extract.

## Data Availability

The original contributions presented in the study are included in the article, further inquiries can be directed to the corresponding author.
